# Validation is the central challenge for generative social simulation: a critical review of LLMs in agent-based modeling

**DOI:** 10.1007/s10462-025-11412-6

**Published:** 2025-11-18

**Authors:** Maik Larooij, Petter Törnberg

**Affiliations:** University of Amsterdam, Amsterdam, The Netherlands

**Keywords:** Systematic literature review, Agent-based modeling, Generative agents, Autonomous agents, Large language models, Multi-agents, Validation

## Abstract

Recent advances in Large Language Models (LLMs) have revitalized interest in Agent-Based Models (ABMs) by enabling “generative” simulations, with agents that can plan, reason, and interact through natural language. These developments promise greater realism and expressive power, but also revive long-standing concerns over empirical grounding, calibration, and validation—issues that have historically limited the uptake of ABMs in the social sciences. This paper systematically reviews the emerging literature on generative ABMs to assess how these long-standing challenges are being addressed. We map domains of application, categorize reported validation practices, and assess their alignment with the stated modeling goals. Our review suggests that the use of LLMs may exacerbate rather than alleviate the challenge of validating ABMs, given their black-box structure, cultural biases, and stochastic outputs. While the need for validation is increasingly acknowledged, studies often rely on face-validity or outcome measures that are only loosely tied to underlying mechanisms. Generative ABMs thus occupy an ambiguous methodological space—lacking both the parsimony of formal models and the empirical validity of data-driven approaches—and their contribution to cumulative social-scientific knowledge hinges on resolving this tension.

## Introduction

Human societies emerge from countless interactions of individuals—yet, social scientific methods tend to focus on either aggregate patterns or individual decision-making, while neglecting the central question of the relationship between the two (Byrne and Callaghan 2022). Agent-Based Models (ABMs) promise a means of changing this, by enabling researchers to simulate how macro-level patterns emerge from micro-level interactions. By modeling individuals as autonomous agents with heterogeneous characteristics and adaptive behaviors, ABMs offer a way to capture the decentralized, dynamic, and non-linear nature of social systems (Holland 1992). This makes them particularly well-suited for studying phenomena such as collective action, innovation diffusion, political polarization, segregation, and financial instability—domains where emergent dynamics are difficult to analyze using traditional methods. ABMs also enable counterfactual experimentation, allowing researchers to explore how different policy interventions or structural shifts might reshape social outcomes.

Despite their theoretical advantages, however, ABMs have historically struggled to achieve widespread adoption within the social sciences. Two key limitations are often cited.

First, the models have been criticized for tending to oversimplify human behavior by representing individuals as simple rule-followers or optimizers (Xi et al. 2025; Epstein 2012). While this approach has been necessary to make computational implementation feasible, it often fails to capture the complexity of human decision-making, which is characterized by more complex reasoning, story-telling, as well as learning, emotions, social norms, and cognitive biases (Törnberg and Uitermark 2025). Some proponents argue that this simplicity is a feature rather than a flaw: by isolating minimal behavioral assumptions, ABMs can illuminate how complex outcomes arise from simple rules—a core principle of complexity science (Holland 1992). Still, the gap between modeled behavior and human cognition remains a source of skepticism.

Second, ABMs face persistent concerns around empirical grounding. Unlike statistical models that rely on established estimation techniques, ABMs are often constructed from numerous assumptions about agent behavior, making calibration and validation difficult (Windrum et al. 2007; Heath et al. 2009). The absence of standardized practices for validating ABMs has raised concerns about their reliability, reproducibility, and generalizability (Fagiolo et al. 2007; Helbing 2012). As each model is often tailor-made for a specific context, comparisons across models are challenging, and cumulative knowledge building is hindered (Ormerod and Rosewell 2006). Scholars have long stressed that simulation models must be rigorously validated to make meaningful contributions to scientific understanding (Naylor and Finger 1967)—yet many ABMs remain at the level of illustrative “toy models.” Without the constraints imposed by tethering variables to empirical data, model complexity can obscure rather than illuminate—as von Neumann famously quipped, “with four parameters I can fit an elephant, with five I can make him wiggle his trunk”.

ABMs have therefore existed in a fundamental tension between the contradictory aims of realism and explainability, and between their inherent versatility and the demands of validation, calibration, and comparability. These tensions have limited their adoption in mainstream social science, particularly as the rise of data-rich computational social science and machine learning in the 2010s shifted focus toward empirically grounded, predictive approaches (Conte and Paolucci 2014).

Recently, however, ABMs have experienced an unexpected resurgence. The advent of Large Language Models (LLMs)—which can generate fluent, context-aware natural language, mimic human reasoning, and exhibit goal-directed behavior—has led researchers to explore their integration into ABMs. This new class of models, often referred to as “Generative ABMs” (GABMs) or “generative social simulation”, leverages LLMs to simulate human-like agents who can plan, reason, and interact via language (Törnberg et al. 2023; Park et al. 2023; Larooij and Törnberg 2025). LLM-driven agents promise to overcome the behavioral limitations of traditional ABMs, offering more nuanced and expressive simulations grounded in linguistic, cultural, and contextual knowledge (Guo et al. 2024). Such models have been applied across a wide array of domains—including policy debates (Chan et al. 2024; Hua et al. 2023), economic behavior (Horton 2023; Li et al. 2024a, 2024b), epidemic modeling (Williams et al. 2023), online discourse (Park et al. 2022; Gao et al. 2023), psychology (Aher et al. 2023), gaming ((FAIR) et al. 2022), software engineering (Qian et al. 2023), and embodied agent interaction (Mandi et al. 2024).

While generative ABMs appear to address the long-standing issue of behavioral realism, their implications for the second major limitation—rigorous validation and calibration—remain uncertain. Indeed, the integration of LLMs may introduce new challenges: their black-box nature, stochasticity, and cultural biases can make it even more difficult to understand, replicate, or empirically ground simulation results.

This paper carries out a systematic review of the rapidly growing field of generative ABMs to examine whether and how modelers have dealt with the central challenge of validation. The paper situates generative ABMs in the longer history of ABMs and the long-standing challenges of validation and calibration, and discusses the potential and pitfalls of generative simulations. The review has a dual aim. First, it provides a descriptive map of what generative ABMs currently simulate (RQ1) and how they are validated (RQ2). Second, it evaluates whether those validation practices meet a minimal standard of operational validity relative to stated modeling purposes (RQ3).

We find that while generative ABMs open exciting new avenues for simulation research and behavioral modeling, they remain constrained by the enduring challenge of validation. In many cases, the addition of LLMs exacerbates rather than resolves this issue, complicating interpretability, standardization, and empirical grounding (Zhao et al. 2024). Nevertheless, the expressive potential of generative simulations makes them a promising area for methodological innovation—promising the potential of a valuable, if currently unsettled, contribution to the evolving toolkit of computational social science.

## Agent-based modeling in the social sciences

Imagine a murmuration of starlings. Collectively, they form a fluid cloud, moving as a single organism. Yet, there is no “group mind” or leader orchestrating the flight. Each bird simply responds to its neighbors, who in turn respond to it—producing a fluid, nonlinear pattern that appears both choreographed and organic. Modeling this movement from a global perspective—as, say, a set of interacting variables—would be impossible (Macy and Willer 2002). But, as early computer modelers discovered in the 1980s, if we model the individual birds, then the dynamics of the murmuration will simply *emerge* from the bottom-up through the aggregation of local interactions (Reynolds 1987).

ABMs take the same approach to studying human social dynamics, treating social systems as consisting of ‘agents’ that perceive their environment, interact and take actions—and casting society as a form of murmuration: a global pattern that emerges through individual interaction (Russell and Norvig 2016). Traditional sociological methods view society as a hierarchical system where institutions and norms shape individual behavior from the top down, captured through a set of variables. ABMs, by contrast, explore how complex social patterns emerge from decentralized interactions among many agents. Instead of imposing top-down explanations, ABMs tend to view social structures, norms, and institutions as emerging from the interactions of individuals (Epstein and Axtell 1996). This imagines human groups as similar to ant-hills or bird flocks: they are *complex systems*; nonlinear, path-dependent, and self-organizing. ABMs enable studying how these dynamics shape social phenomena, as macro-patterns unexpectedly emerge from underlying micro-level mechanisms—which is often impossible to achieve using conventional methods (Törnberg and Uitermark 2025). At the same time, these models offer only potential mechanisms: they can provide *sufficient* explanations, not *necessary* ones, as the same macro-phenomenon can be explained by several different micro-level mechanisms.

ABM as a social scientific method traces back to the early 1970s, with Thomas Schelling’s influential models of social segregation (Schelling 1971). The method saw rapid growth as the rising availability of computers made modeling accessible to a broad range of researchers. In the 1990s and early 2000s, ABMs grew substantially, together with the broader field of complexity science. The method developed its own journals (e.g., the Journal of Artificial Societies and Social Simulation, JASSS) and scientific association (The European Social Simulation Association, ESSA).

From 2010 onward, however, ABM and complexity science stalled, as it faced limited adoption within the social sciences, and was challenged by the growth of Computational Social Science and its emphasis on data analysis over generative explanation (Conte and Paolucci 2014).

ABMs faced growing criticism for being empirically untethered to the systems being represented, reflecting the ad hoc intuitions of the modeler rather than the dynamics of the real world. The underlying rules governing the actions of the agents would often be simply assumed by the modeler based on what seemed to them to make sense, often without a clear link to neither empirical data nor existing social scientific theory (Windrum et al. 2007). While such an approach may have been acceptable in an era of data scarcity, the growing availability of digital data in the 2010 s intensified demands that ABMs should be calibrated and validated against the real world (Naylor and Finger 1967; Ormerod and Rosewell 2006; Ziems et al. 2024; Macy and Willer 2002). Critics revived long-standing arguments that models that have not been subjected to proper validation are “void of meaning” and contribute nothing to the understanding of the simulated system (Naylor and Finger 1967). Without rigorous empirical grounding, ABMs remain mere toy-models or illustrations, limiting their usefulness for contributing to social science research, and their capacity to influence policy.

As journals increasingly began requiring ABMs to be calibrated and validated against real-world data, this often turned out to be a tall order. As ABMs seek to capture emergent outcomes from large numbers of interacting agents, they are themselves complex systems (Silverman et al. 2009). While this property is necessary for the models to capture social complexity, it also means that the models display many of the pernicious properties that make complex systems challenging to study: the models are often mathematically chaotic, and hence sensitive to initial conditions (Bertolotti et al. 2020), making them challenging to reproduce. They are moreover often high-dimensional, with their many degrees of freedom resulting in the so-called ‘curse of dimensionality’ (De Marchi 2005). When models are untethered to empirical data, this dimensionality furthermore means that ABMs are computationally costly to examine: the agent interactions tend to scale quadratically with the number of agents, whereas sensitivity analyses scale exponentially with the number of parameters.

The flexibility and lack of standardization means that few design constraints are imposed by ABMs. As a result, every model is specifically tailored for its intended purpose, making models difficult to compare (Fagiolo et al. 2007). The lack of comparability represents an impediment to the type of universal methodologies and benchmarks that are crucial for achieving cumulativity of research findings, as it is otherwise unclear how the findings of one model speak to those of another model, let alone to dynamics in the real world. As a result, the field of ABMs faced a “replication crisis”, as studies found that the models could almost never be replicated—and that some of the findings in the field turned out to be the results of software bugs (Wilensky and Rand 2007).

The lack of empirical grounding implies the need for the models to be highly convincing representations of the systems they simulate. However, social scientists have remained skeptical of the capacity of the often simple rule-based models to represent human behavior. The theoretical descriptions of ABMs as consisting of intelligent, adaptive, and goal-seeking agents that dynamically shape and respond to their environment while taking into account past events (Epstein and Axtell 1997) stood in often stark contrast to their realities as simple rule-followers based on a series of “if-then” statements or optimization. While some models sought to pursue more complex representation of human behavior, the emphasis has remained on simplicity. While complexity scientists would argue that it is precisely this simplicity that makes these models useful for exploring social complexity (Epstein and Axtell 1996), many social scientists argued that the models fail to encompass the full reality of human decision-making, in particular as the role of beliefs, memories, story-telling, and past interactions are rarely accounted for (Sargent 2010). While ABMs do represent improvements over the atomized perfect optimizers of neoclassical theory (Törnberg 2018), they were criticized for remaining part of the same broader methodologically individualist paradigm (Byrne and Callaghan 2022).

ABMs have furthermore proven challenging to integrate with existing social scientific theory. In part due to their simplicity, the explanations offered by ABMs often seemed to be fundamentally at odds with existing social scientific explanations, as they tend to cast any social phenomenon as simply ‘emerging’ from bottom-up interactions (Macy and Willer 2002). Such reductive explanations tend to come with a hint of the same political biases as neoclassical models, through the lens of which the market appears as ‘natural’, while the top-down role of institutions is viewed as imposing unnatural constraints (Baker 2022; Foucault 2008). The widely lauded Schelling (Schelling 1971) segregation model, for instance, seemed to suggest that segregation naturally “emerged” from an innate mutual dislike of the opposing group, while disregarding dominant explanations like structural racism, white flight, and red-lining—thereby also seeming to eradicate the possibility of collective solutions (Törnberg and Uitermark 2025).

These challenges limited the adoption of ABMs within the social scientific mainstream. With the growing capacities of machine learning and AI to produce insights from the increasingly abundant digital social data, the social data analytics and computational social science came to overshadow ABMs from the 2010s onward (Conte and Paolucci 2014).

### Generative ABMs

The emergence of Large Language Models (LLMs) has recently brought an unexpected change in the fortunes of ABMs. While ABM research had already begun exploring deep learning approaches to modeling human behavior (Xi et al. 2025), LLMs introduce a fundamentally different paradigm. Trained to generate and interpret natural language, they can in many respects be viewed as ready-made models of human behavior and cognition. Unlike conventional ABMs (Zhao et al. 2023), these so-called “generative agents” are built on vast corpora of textual data, endowing them with internal world models that enable generalization to new situations and imitation of human-like reasoning. Moreover, generative agents are capable of producing and interpreting natural language in ways that can be indistinguishable from human communication (Jones et al. 2025).

These capabilities were first demonstrated in a 2023 study that used LLMs to simulate human behavior in a Sims-like virtual environment, where generative agents began to exhibit human-like behavior and complex social dynamics (Park et al. 2022). This study catalyzed rapid growth in the field of generative ABMs, prompting a wave of reviews aimed at mapping the contours of this emerging research area (Guo et al. 2024; Xi et al. 2025; Wang et al. 2024a, b; Cheng et al. 2024; Mou et al. 2024a, 2024b).

In generative ABMs, agents are typically assigned distinct personas–comprising demographic, personal, and social characteristics—that may be hand-crafted, AI-generated, or derived from empirical data. Agents often possess memory modules for storing and retrieving relevant information, and planning modules that enable them to formulate and pursue self-generated plans. These cognitive components allow agents to interact with their environments by changing states (e.g., setting a toilet to ‘occupied’), updating internal beliefs, or communicating with other agents.

Generative agents therefore appear to address one of the most persistent criticisms of ABMs: their lack of behavioral realism. By enabling simulations with agents that can remember, reason, argue, and converse in ways that appear convincingly human (Park et al. 2022), they have generated considerable enthusiasm in the modeling community.

At the same time, while generative ABMs appear to address the issue of realism, it remains an open question how and whether generative ABMs resolve the challenge of validation. Although generative agents produce behavior that appears more realistic than that of their rule-based antecedents, it remains to be demonstrated that such behavior aligns with that of real-world individuals and groups. Moreover, the reliance on LLMs introduces additional complications. In what follows, we outline three interrelated ways in which the use of LLMs potentially bring additional challenges for validating agent-based models.

First, LLMs are fundamentally *black-box* models: their capacities are emergent, and it is virtually impossible to determine why a particular input yields a particular output. These models are based on large-scale neural networks, with behavior shaped by billions of parameters (Zini and Awad 2022). As the complexity of these networks increases, our ability to interpret their internal decision-making processes diminishes. Furthermore, LLMs are inherently stochastic—the same input can produce different outputs across runs—posing additional challenges for validation and reproducibility.

Second, the models cannot be simply assumed to accurately represent social groups or impersonate human behavior. The models’ outputs differ in fundamental ways from human text, often exhibiting exaggerated politeness, verbosity, and excessive eloquence, and have proven challenging to finetune to reproduce text with human-likeness (Pagan et al. 2025). When models are given descriptions of individuals to impersonate, studies have found that the models often misrepresent groups and their attributes, often engaging in exaggerated stereotypes rather than accurate representations (Boelaert et al. 2025; Cheng et al. 2023). This issue is closely tied to the widely debated problem of bias in LLMs (Ferrara 2023; Abid et al. 2021; Kotek et al. 2023; Ghosh and Caliskan 2023). In particular, two forms of bias present major challenges for the validation of generative ABMs: *social bias* and *selection bias* (Navigli et al. 2023). Social bias refers to the reproduction of discrimination, stereotypes, or prejudices toward certain groups, resulting in agents that reflect problematic portrayals rather than accurate representations. Selection bias, by contrast, stems from the composition of the training corpus: LLMs may draw on historically contingent or unrepresentative texts, leading them to reproduce past events or complete social network interactions based on prior knowledge, rather than generating behavior dynamically as intended by the model design. Since the models have been trained on scientific literature, what may appear as emergent dynamic can instead stem from a form of “data leakage”, as the models are simply reproducing phenomena identified in previous studies (Barrie and Törnberg 2025).

Third, as LLMs are probabilistic next-word predictors, they possess no internal mechanism to validate the correctness of their outputs. They may hence generate outputs that are factually incorrect or nonsensical—a phenomenon often referred to as “hallucination” (Huang et al. 2023). This problem is compounded when models are placed in situations that are substantially different from those represented in their training data. In such out-of-distribution contexts, model behavior can become erratic or inconsistent, producing outputs that deviate sharply from plausible or expected patterns. This poses a particular challenge for social scientific applications in which generative agents are used to simulate scenarios with little or no historical precedent, as uncertainty in the models’ responses may be driven as much by the unfamiliarity of the setting as by the models’ tendency to hallucinate.

These challenges do not imply that validation in generative ABMs is inherently unattainable. Rather, they point to issues that must be addressed by the field, and highlight the particular conditions under which robust validation strategies must be developed.

### Validation in ABMs

Before turning to a review of how validation has been approached in the emerging field of generative ABMs, we first draw on the broader literature on ABM validation to outline commonly discussed validation types. Validation refers to the process of assessing the extent to which a model accurately represents the real world (Ormerod and Rosewell 2006). As Sargent (2010) notes, the goal of validation is not to replicate the target system in its entirety, but rather to establish *operational validity*: “determining whether the simulation model’s output behavior has the accuracy required for the model’s intended purpose over the domain of the model’s intended applicability”. Models are always abstractions, and it is necessary to bracket part of the system being modeled. ‘Realism’ in the broad sense is, in other words, neither achievable nor desirable.

A first distinction in the literature is between *internal* and *external* validation. External validation relies on sources outside the model for assessment—such as empirical data, well-documented behaviors, or human judgment—whereas internal validation focuses on the coherence and behavior of the model itself. The latter can involve observing the system’s dynamics, reasoning about emergent outcomes, or performing sensitivity analyses to evaluate the robustness of results (Christopher Frey and Patil 2002).

A second key distinction is between *subjective* and *objective* validation. Subjective approaches include techniques such as expert judgment or qualitative assessment of system behavior through traces, visualizations, and performance indicators. A notable example is the Turing Test, in which human evaluators assess whether they can distinguish between model-generated and human-generated outputs (Turing 2009). Objective validation, by contrast, involves comparison with empirical data or established models using statistical methods. One of the earliest contributions to this approach proposed a multi-stage validation framework: first, develop model assumptions; second, empirically test those assumptions where possible; and third, compare the model’s input–output relationships to those observed in the real system (Naylor and Finger 1967).

For the evaluative discussion (RQ3), we use a minimal yardstick for good validation practice consistent with operational validity: (i) purpose alignment—the validation target must correspond to the mechanism or outcome the model is meant to inform; (ii) external grounding—evidence based on human-generated data or pre-registered experimental benchmarks, rather than face-validity alone; and (iii) robustness—results reported across multiple runs and, where feasible, limited sensitivity checks for key parameters.

## Method

**Fig. 1:**
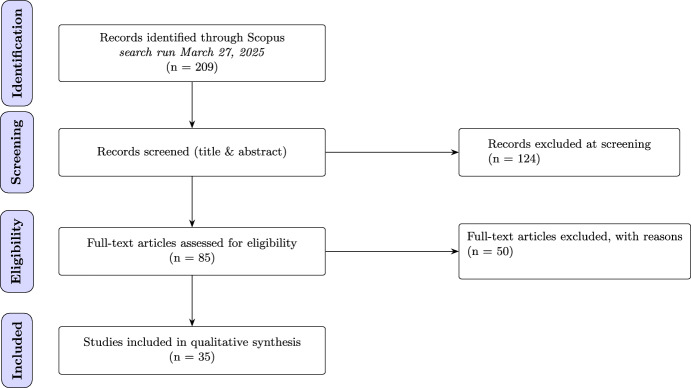
PRISMA 2020 flow diagram of study selection (Scopus search conducted March 27, 2025)

To identify relevant publications on generative ABM, we conducted a systematic literature search using *Scopus*, a widely used bibliographic database (see Fig. 1). The search was performed on March 27, 2025, with the goal of capturing recent research combining LLMs and ABMs across multiple disciplines, including computer science, artificial intelligence, and computational social science. We developed a Boolean query that reflects key concepts in this emerging field, targeting the TITLE, ABSTRACT, and KEYWORDS fields to balance breadth and precision:
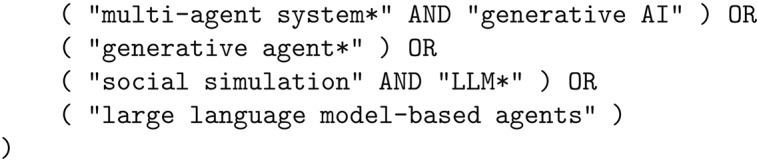


This initial query yielded 209 papers. To expand the search and ensure comprehensive coverage, we employed backward snowballing (Wohlin 2014), reviewing the reference lists of the included papers, after having filtered the initial papers based on the procedure below. Our initial search included several survey papers of the field, which were particularly useful for identifying additional publications (Guo et al. 2024; Xi et al. 2025; Wang et al. 2024a,2024b; Cheng et al. 2024; Mou et al. 2024a, 2024b).

We applied a two-stage filtering process to determine the final set of papers for inclusion:

1. Title and abstract screening: Irrelevant papers were excluded based on a preliminary review of titles and abstracts.
2. Full-text review: The remaining papers were assessed in detail to determine methodological rigor and direct relevance to our research questions.

We included studies that met all of the following eligibility criteria:

- The use of large language models (LLMs) to generate agent behavior.
- The inclusion of multiple agents.
- The presence of interactions between agents, where behavior depends on the state of the system or on other agents.
- An explicit aim to simulate or reproduce human social behavior.

We excluded studies that:

- Focused primarily on task completion rather than social simulation.
- Did not aim to replicate or model human behavior.
- Were not published in English.
- Were review or survey articles rather than original research.

These criteria led us to exclude papers in which agents acted in isolation without mutual influence, such as Feng et al.’s Feng et al. (2024) study on electric vehicle charging behavior, Ren et al. (2024a), (2024b) work on web search simulation using agents with varying personalities, and Park et al. (2024) framework for simulating realistic individuals agents based on interviews and survey data. We also excluded studies that used generative agents for synthetic data generation (Argyle et al. 2023), and those focused on infrastructure or frameworks for generative ABMs rather than on modeling human behavior—such as CAMEL (Li et al. 2023a), (2023b), MetaGPT (Hong et al. 2024), CGMI (Jinxin et al. 2023), and AutoGen (Wu et al. 2024a, b). After applying these criteria, 35 papers remained.

We conducted an in-depth analysis of the selected papers, with a focus on sections related to model design, evaluation, and limitations. Each paper was manually reviewed, and relevant data were extracted into a structured database covering key characteristics, including model purpose, agent architecture, interaction mechanisms, and validation strategies.

The coding was conducted by a single coder. We acknowledge this as a potential limitation, as the absence of inter-coder agreement checks ma

**Table 1:** Classification of the reproduced social phenomena for the 35 papers

	Profile Alignment	Emotion	Conversation/Content	Social Awareness	Decisions/Reasoning	Opinion/Attitude	Network Propagation	Network Structure	Social Dynamics
Park et al. (2023)									
Hua et al. (2023)									
Park et al. (2022)									
Gao et al. (2023)									
De Marzo et al. (2023)									
Wang et al. (2023)									
Kaiya et al. (2023)									
Zhang et al. (2024a), (2024b), (2024c)									
Chen et al. (2024)									
Williams et al. (2023)									
Altera.al et al. (2024)									
Li et al. (2023a), (2023b)									
Li et al. (2024a), (2024b)									
Zhang et al. (2024a), (2024b), (2024c)									
Liu et al. (2024a), (2024b), (2024c)									
Zhou et al. (2024)									
Zhang et al. (2024a), (2024b), (2024c)									
Li et al. (2025)									
Xie et al. (2024)									
Ren et al. (2024a), (2024b))									
He et al. (2024)									
Wu et al. (2024a), (2024b)									
Jarrett et al. (2023)									
Yu et al. (2024)									
Chuang et al. (2024)									
Liu et al. (2024a), (2024b), (2024c))									
Mou et al. (2024a), (2024b)									
Yang et al. (2024)									
Sreedhar and Chilton (2024)									
Liu et al. (2024a), (2024b), (2024c))									
Gu et al. (2025)									
Orlando et al. (2025)									
Wang et al. (2025)									
Ferraro et al. (2025)									
Zheng and Tang (2025)									
Total	8	2	10	5	7	2	8	5	10
		Individual				Group			

y introduce subjectivity or bias. That said, this limitation may be less consequential in the present context, as the goal of the review is to classify and compare validation strategies rather than interpret ambiguous qualitative data. While every effort was made to ensure consistency and transparency in interpretation, future research may benefit from involving multiple coders and reporting inter-coder reliability metrics.

The purpose of this review is to provide an overview of the social phenomena that generative simulations are used to model, and to examine how researchers approach the challenge of model validation. Our analysis is guided by the following research questions:

1. **RQ1:** What types of social phenomena are generative simulations used to model?
2. **RQ2:** What validation strategies are reported in the literature?
3. **RQ3:** Are the reported validation strategies adequate for the model’s stated purpose in terms of achieving operational validity?

To answer RQ1, we distinguish between simulating *individual* and *group* behavior. Papers that seek to reproduce individual behavior focus on the actions of a single agent, for example if decisions are congruent with a given profile. Other papers search for group behavior at the macro-level of the simulation, like the formation of the network through connections, or the propagation of information through the network. For individual behavior, we further distinguish between the following aspects of the simulated phenomenon, which were identified inductively through an initial analysis:

1. Alignment with profile: the alignment of the agent’s actions with their personality, memories and profile, as well as the specific situation.
2. Emotion: the emotions expressed by the agent given a specific situation or action.
3. Conversations and content generation: the communication and dialogue between agents.
4. Social awareness: the awareness of others and of social norms.
5. Decision making and reasoning: the decision making and reasoning capabilities.
6. Opinion and attitude: the opinion or attitude towards some event, and possibly the shift in opinion or attitude.

For group behavior, we used the following three categories:

1. Network propagation: the propagation of something through the network, such as (mis)information, emotion, attitudes, or cultural elements.
2. Network structure: the structure of the network, for example the network degree distribution, density or some other network structure attribute.
3. Social dynamics: phenomena that emerge from individuals actions, for example a ’herd effect’

Some papers focus on multiple phenomena are thus given multiple categorizations. A paper is classified as focusing on a certain type of behavior only if the paper sets out to model that behavior, thus excluding observed phenomena that are noted only as an aside. For example, Kaiya et al. (2023) set out to measure social reasoning, but note some human-like emotions and reactions.

To answer RQ2, we analyzed the reviewed studies to identify recurring strategies for validating generative ABMs. Rather than applying a predefined typology, we took an inductive approach: we examined how different studies approached validation and grouped them based on shared features and logics.

In line with the existing literature, we separate between *internal* and *external* validation, as well as between *subjective* and* objective* validation. These categories should not be understood as strict classification axes. Instead, we use them as heuristic labels to characterize the nature of each technique, helping to highlight variation in how validation is approached.

Based on our analysis, we identify five main categories of validation used in the current literature:

- Validation based on human(-like) judgment (typically External/Subjective): involves using input from researchers, experts, crowd-workers, or LLMs to assess the plausibility of model behavior.
- Validation against well-known social patterns (typically External/Subjective): compares model outcomes to widely recognized patterns in social behavior, albeit not through quantitative benchmarks.
- Validation against similar models (can be External/Subjective or Objective): involves comparison with earlier models, either qualitatively (e.g., plausibility of dynamics) or quantitatively (e.g., similarity of outputs).
- Validation against human-generated data (typically External/Objective): compares model outputs to empirical data such as social media behavior, annotated datasets, or experimental results.
- Validation based on internal consistency (Internal/Objective): evaluates the model’s internal logic and robustness, for example through sensitivity analysis or parameter sweeps.

To address RQ3, we evaluate whether each study’s reported validation is sufficient to establish operational validity—that is, whether the validation approach meaningfully supports the model’s intended purpose and target system. Specifically, we examine whether the validation evidence plausibly links to the mechanisms or outcomes the model is designed to explain, and seek to identify any validity issues—for example, validations that rely on weakly coupled metrics or single-run results without robustness checks. We do not reanalyze data or apply new evaluation criteria; instead, we synthesize these study-level judgments to identify patterns in current practice and highlight where existing approaches appear adequate or where significant gaps persist.

## Results

We identified 35 papers that met the eligibility criteria. Table 1 provides a full classification. The most common topics were content generation and conversation, social dynamics, and network propagation. While 21 papers focus on a single category, the remainder engage with multiple themes–resulting in an average of 1.63 categories per paper.

### Target systems of simulation

This section reviews the types of social phenomena modeled in generative ABMs, organized by thematic category. For each, we describe the modeling aim and the validation approaches employed.

#### Profile alignment

A key modeling aim in many generative ABMs is to capture how agents’ actions reflect their memories, personality traits, and past experiences—an aspect we refer to as *profile alignment*. However, validating profile alignment presents a major challenge. It is rarely clear what constitutes the “correct” behavior for a given agent in a specific context, and access to ground truth is typically unavailable, as it would require comparison to the real individuals being simulated.

Several studies have attempted to address this challenge through various forms of “believability” assessment. Park et al. (2023) validate agent behavior by interviewing agents about their self-knowledge, memory, plans, and reflections, and evaluating whether their responses align with their personality, memory, and environmental context. Yu et al. (2024) build on this approach, assessing the believability of their Affordable Generative Agents using similar methods. Wang et al. (2025) likewise evaluate whether agent behavior and memory function are perceived as believable.

Other studies adapt the notion of profile alignment to specific domains. Hua et al. (2023) simulate geopolitical interactions and use expert assessments to evaluate whether agents’ actions—such as military decisions–are congruent, stable, and rational in relation to their national profiles. Altera.al et al. (2024) deploys agents in a Minecraft environment, validating profile alignment by observing whether agents specialize into roles that correspond to their predefined traits and whether their actions reflect those roles. Jarrett et al. (2023) introduce "digital representatives" of real individuals and assess alignment based on how closely these agents reflect the preferences of the individuals they are meant to represent in collective decision-making contexts. Liu et al. (2024a), (2024b, (2024c) examine whether agents’ shopping decisions align with their profile characteristics, contextual factors, and known expectations or constraints.

#### Emotional alignment

Two of the included studies focus on modeling and validating emotional responses in generative agents. Gao et al. (2023) aim to predict users’ emotional reactions to specific events on social media, categorizing responses into three levels: calm, moderate, and intense. Wang et al. (2023) develop a broader platform that incorporates basic needs, social relationships, and emotional states. They validate emotional alignment by comparing agents’ expressed emotions during various activities to human-annotated labels.

#### Conversations and content generation

LLMs enable simulation of systems in which individuals engage in conversation, such as social media platforms. Many studies validate these interactions by comparing the generated content to that of human participants, often by testing whether human coders are able to distinguish between synthetic and human conversations. Park et al. (2022), for instance, develop a synthetic version of Reddit, and test whether human participants are able to tell which conversations are simulated. Yu et al. (2024) adopt a similar validation strategy in a virtual town setting.

Other studies employ quantitative methods to assess textual similarity. Gu et al. (2025) compute cosine similarity between synthetic and human-generated text embeddings, while Gao et al. (2023) augment this with perplexity scores to measure fluency. Ferraro et al. (2025) examine whether agents generate keywords, interests, and content distributions similar to human users. Mou et al. (2024a), (2024b) focus on the stance of generated posts and their thematic alignment with real-world Twitter data. They further categorize content types—such as “call to action,” “opinion sharing,” and “third-party references”—and compare these distributions to real-world Twitter data.

In contrast, some studies emphasize the *humanness* of conversations rather than content similarity. Wang et al. (2023) evaluate conversational realism based on whether dialogue leads to increased social closeness between agents. Zhang et al. (2024a), (2024b), (2024c) validate generated dialogues by assessing coherence with scenario context, character consistency, and script quality. Zhang et al. (2024a), (2024b), (2024c) take a more fine-grained approach, rating dialogues on dimensions such as naturalness, empathy, and interestingness, as well as the agent’s ability to select appropriate dialogue strategies. Similarly, Liu et al. (2024a), (2024b), (2024c) compare conversations based on expressiveness and perceived naturalness relative to human benchmarks.

#### Social awareness and social intelligence

Social awareness refers to the collective consciousness and shared norms within a society (Schlitz et al. 2010), while social intelligence concerns an agent’s ability to interpret and respond appropriately to the actions and mental states of others. In generative ABMs, validation in this area typically focuses on whether agents can adhere to social norms, recognize social cues, and reason about others’ beliefs or intentions.

Altera.al et al. (2024) evaluate agent’ capacities for both self-awareness and understanding of others, testing whether agents can correctly infer the sentiments of peers and adjust their behavior accordingly. Li et al. (2023a), (2023b) explore whether agents possess a form of *Theory of Mind*—the ability to reason about the unobservable mental states of others.

Zhou et al. (2024) propose a benchmark, SOTOPIA-EVAL, to evaluate social intelligence across diverse scenarios. The benchmark includes dimensions such as believability, social rule adherence, relationship management, and strategic use of information (e.g., revealing or withholding secrets). Similarly, Xie et al. (2024) assess whether agents exhibit trust-based behavior in interactive economic games like the Trust Game and the Dictator Game.

Ren et al. (2024a), (2024b) shift the focus to norm formation, examining how social norms emerge and are incorporated into agent reasoning and decision-making in a simulated sandbox society.

#### Decision-making and reasoning

Simulating human decision-making and reasoning processes is a central objective in many generative ABMs. A common validation approach is to compare agent behavior against real-world decisions or theoretically expected outcomes.

Gao et al. (2023) examine agents’ decisions on social media–such as sharing, posting, or remaining inactive–and compare these choices with those of actual users. Kaiya et al. (2023) evaluate agents’ autonomy and social reasoning across three narrative-driven scenarios: a murder mystery, a high school activity fair, and a situation requiring aid to a patient. Williams et al. (2023) assess whether agents can make contextually appropriate decisions–such as staying at home or quarantining–in a simulated pandemic resembling COVID-19.

Other studies test agents’ reasoning under more abstract or rule-based conditions. Altera.al et al. (2024) investigate agents’ capacity to reason about societal norms and rules. Wu et al. (2024a), (2024b) explore whether agents can make adaptive decisions in the absence of explicit instructions, focusing on competitive scenarios that require cooperation to achieve optimal outcomes. Sreedhar and Chilton (2024) replicate the Ultimatum Game to examine agents’ ability to exhibit strategic reasoning comparable to human participants.

Finally, Liu et al. (2024a), (2024b), (2024c) simulate consumer decision-making, validating whether agents’ shopping behaviors align with expectations given their profile, product context, and historical purchase patterns.

#### Opinions and attitudes

A smaller set of studies use generative agents to simulate human attitudes or opinions, typically validating whether agents’ responses to events or stimuli reflect plausible human reactions. Gao et al. (2023) evaluate agents’ attitudes—classified as positive or negative—toward social media posts by comparing them to real-world user responses. Wang et al. (2023) assess whether agents can predict whether certain activities fulfill basic human needs such as fullness, sociability, enjoyment, health, and energy, thereby measuring agents’ evaluative responses to events and their effects on well-being.

#### Network propagation

Network propagation is a major area of focus in generative ABMs, with 8 out of 29 reviewed papers including some form of validation related to the spread of information, emotions, attitudes, or norms. These validations typically compare the simulated diffusion patterns to known behavioral dynamics observed in real-world social networks.

Park et al. (2023) examine the diffusion of information through agent networks. Gao et al. (2023) go further by measuring the propagation of information, emotion, and attitudes on social media platforms. Several studies simulate the spread of misinformation, including Li et al. (2024a), (2024b) and Liu et al. (2024a), (2024b), (2024c), who use synthetic social media environments to study how false information circulates.

Yang et al. (2024) analyze the spread of various types of information across networks. Altera.al et al. (2024) simulate the diffusion of cultural memes and religious beliefs, while Ren et al. (2024a), (2024b)) model the spread and internalization of social norms within a sandbox society.

#### Network structure

Several studies focus on validating the structure of networks generated through agent interactions, aiming to ensure that emergent network properties resemble those observed in real-world systems. This typically involves assessing whether the characteristics of the network match the properties of real world networks.

De Marzo et al. (2023) evaluate whether their simulated networks exhibit scale-free distributions–a common feature of social media networks. He et al. (2024) examine whether their synthetic platform, *Chirper.ai*, reproduces homophily, the tendency for agents to connect with similar others.

Park et al. (2023) and Yu et al. (2024) analyze the evolution of social networks in sandbox simulations, using metrics such as node degree and network density. Hua et al. (2023) validate the historical plausibility of international alliances and war declarations formed by agents simulating countries. Gu et al. (2025) assess the formation of echo chambers using standard network measures, including modularity, average path length, density, and clustering coefficient.

#### Social dynamics

This final category encompasses papers that aim to reproduce emergent social dynamics–patterns of group behavior arising from individual actions. These include phenomena such as conformity, polarization, norm formation, and collective decision-making.

Zhang et al. (2024a), (2024b), (2024c) observe group-level phenomena such as conformity, consensus formation, and emergent dynamics in their agent society. In a Minecraft-based simulation, Chen et al. (2024) find agents displaying volunteering, conformity, and even destructive behavior as survival strategies. Similarly, Wang et al. (2025) identify both conformity and the emergence of an “information cocoon” effect.

Echo chambers–where agents interact primarily within ideologically homogenous groups–are another recurring focus, studied in several papers (Gu et al. 2025; Ferraro et al. 2025; Zheng and Tang 2025). Chuang et al. (2024) simulate the “Wisdom of Partisan Crowds,” showing how group exposure to average partisan beliefs can shift opinions toward the ground truth, even across political divides.

Mou et al. (2024a), (2024b) analyze attitude dynamics on a simulated Twitter platform (*HiSim*), using statistical measures such as deviation from the mean (bias), standard deviation (diversity), and similarity scores via Dynamic Time Warping and Pearson correlation. Yang et al. (2024) replicate the “herd effect” on Reddit—where early likes or dislikes influence subsequent reactions—and find it to be more pronounced among generative agents than humans, likely due to a lack of critical filtering. Orlando et al. (2025) validate the replication of the “friendship paradox,” the phenomenon where most individuals have fewer friends than their friends do on average.

### Validation techniques

We now turn to the question of how these generative ABMs are validated. Table 2 provides an overview of the validation techniques used across the reviewed studies. For classification purposes, we distinguish between *primary* and *secondary* techniques. Primary techniques represent the core method used for validation in each paper (marked in green), while secondary techniques serve as supplementary evidence (marked in orange).

In the following subsections, we review each validation approach in turn, drawing on specific examples from the literature.

#### Validation based on human or human-like judgment

One of the most frequently used validation strategies involves relying on judgments from either human evaluators or other LLMs. These evaluators fall into three main categories: (1) domain experts (often the authors themselves), (2) crowd-workers, and (3) LLMs used as evaluators.

In the first category, Hua et al. (2023) employ field experts to evaluate the congruence and consistency of agent actions with national profiles in a geopolitical simulation. Li et al. (2025) rely on author judgment to assess the performance of their MetaAgents in matching job-seekers to workflows and roles. Wang et al. (2025) similarly use human raters to evaluate the believability of agent dialogue and memory systems.

Despite ethical and methodological concerns—including issues of low reliability and the potential use of LLMs by workers themselves—crowd-workers remain widely used, often recruited through platforms such as Amazon Mechanical Turk. Park et al. (2023) and Park et al. (2022) employ crowd-workers to evaluate believability and distinguish between synthetic and human-generated conversations. Crowd-sourced judgments are also used to assess Theory of Mind capabilities (Li et al. 2023a, 2023b) and to test whether humans can differentiate between generative and human agents in various tasks (Ren et al. 2024a, 2024b; Zhou et al. 2024; Zhang et al. 2024a, 2024b, 2024c).

A third and increasingly common approach involves using LLMs themselves to evaluate other generative models. While this introduces obvious circularity and has been criticized as methodologically problematic, it has become increasingly widespread as means of validation, likely motivated by simplicity and low costs. For instance, Zhou et al. (2024) and Zhang et al. (2024a), (2024b), (2024c) use GPT-4 to assess realism and coherence in agent conversations. Jarrett et al. (2023) use an LLM to compare model-generated critiques with ground-truth responses. Liu et al. (2024a), (2024b), (2024c) combine human and GPT-4 evaluation to assess believability, social influence, and linguistic quality. Similarly, Yu et al. (2024) use GPT-4 to distinguish AI-generated responses from human ones—despite prior research indicating that LLMs may be unreliable for this task (Bhattacharjee and Liu 2024).

#### Validation against well-known social patterns

Another common validation approach involves assessing whether simulated dynamics align with well-established social patterns. Unlike empirical validation against specific datasets, this strategy relies on comparing model outputs to broadly recognized behaviors and structures found in prior research. Data leakage represents a serious risk for such validation approaches, as the previous research likely was part of the training data and the LLM may simply be reproducing published results (Barrie and Törnberg 2025).

Some studies focus on easily quantifiable structural patterns. De Marzo et al. (2023), for example, evaluate whether the generated networks exhibit scale-free degree distributions–a typical property of social media networks. Li et al. (2024a), (2024b) show that agents’ propensity to share fake news, influenced by personality traits and profile characteristics, mirrors findings from previous behavioral studies. Liu et al. (2024a), (2024b), 2024c) similarly confirm that fake

**Table 2:** Classification of the validation techniques for the 35 papers

	Human(-like) Judgment	Social Patterns	Other Models	Human Generated	Internal Consistency	Other Models
Park et al. (2023)						
Hua et al. (2023)						
Park et al. (2022)						
Gao et al. (2023)						
De Marzo et al. (2023)						
Wang et al. (2023)						
Kaiya et al. (2023)						
Zhang et al. (2024a), (2024b), (2024c)						
Chen et al. (2024)						
Williams et al. (2023)						
Altera.al et al. (2024)						
Li et al. (2023a), (2023b)						
Li et al. (2024a), (2024b)						
Zhang et al. (2024a), (2024b), (2024c)						
Liu et al. (2024a), (2024b), (2024c))						
Zhou et al. (2024)						
Zhang et al. (2024a), (2024b), (2024c)						
Li et al. (2025)						
Xie et al. (2024)						
Ren et al. (2024a), (2024b))						
He et al. (2024)						
Wu et al. (2024a), (2024b)						
Jarrett et al. (2023)						
Yu et al. (2024)						
Chuang et al. (2024)						
Liu et al. (2024a), (2024b), (2024c)						
Mou et al. (2024a), (2024b)						
Yang et al. (2024)						
Sreedhar and Chilton (2024)						
Liu et al. (2024a), (2024b), (2024c)						
Gu et al. (2025)						
Orlando et al. (2025)						
Wang et al. (2025)						
Orlando et al. (2025)						
Zheng and Tang (2025)						
Total (main technique)	12	14	1	12	1	1
Total (secondary technique)	2	6	3	4	3	2
	Subjective			Objective		

Green bullet signifies that the paper uses the technique as a primary validation method, whereas orange marks that it is a secondary technique

political news spreads faster than other types, and that the relationship between Big Five personality traits and misinformation sharing aligns with earlier empirical work. Orlando et al. (2025) validate the presence of the “friendship paradox,” where individuals tend to have fewer friends than their friends do on average.

Other papers rely on more qualitative comparisons, where validation depends on interpretive arguments about the plausibility of model behavior. Zhang et al. (2024a), (2024b), (2024c) and Chen et al. (2024) identify patterns of conformity, consensus formation, and group dynamics that they argue reflect established findings in social psychology. Williams et al. (2023) note that agent responses to a simulated disease outbreak–such as staying home or quarantining–broadly resemble behaviors observed during the COVID-19 pandemic. Similarly, Yang et al. (2024) report that their model reproduces group polarization effects known from social research.

Some studies take a looser approach to validation. Kaiya et al. (2023) and Altera.al et al. (2024) argue for plausibility based on general similarity between simulated and human behavior, without offering rigorous benchmarks. Ren et al. (2024a), (2024b) adopt a case-study strategy, interpreting emergent dynamics as analogous to norm adoption and conflict formation in real societies. Wu et al. (2024a), (2024b) compare agent behavior to human studies in only one of three experimental conditions, noting some similarities in cooperative strategies.

Several studies validate against specific social patterns such as relationship formation and information diffusion. Park et al. (2023) and Yu et al. (2024) claim that their agents’ behavior aligns with expected patterns of social tie formation and message spread. Mou et al. (2024a), (2024b), Zheng and Tang (2025), Ferraro et al. (2025) all highlight the emergence of echo chambers as a known and reproducible social phenomenon. Wang et al. (2025) similarly report signs of information cocoons and conformity effects.

#### Validation against similar models

Generative ABMs can also be validated through comparison with previous models. The catch here is that it is implied that the previous models are already rigorously validated, which circles back to the validation problem that this paper addresses. As a consequence, authors can conclude on the performance compared to the other model, but the alignment with human behavior depends heavily on the quality of the other model. These comparisons again vary in whether it is carried out in a rigorous quantitative way or in a more subjective way that is argued for in narrative form.

Zhang et al. (2024a), (2024b), (2024c) compare the generated conversations of their SpeechAgents with a previous single-agent model. Mou et al. (2024a), (2024b) and Gu et al. (2025) systematically compare their social media simulations with conventional non-generative ABMs. Liu et al. (2024a), (2024b), (2024c) compare the purchasing behavior of their agents to different filtering algorithms and two other agent based approaches. This system is in turn used by Wang et al. (2025) in a comparison with their approach to recommender agents.

Yu et al. (2024) offers an example of a more subjective comparison, comparing their model with Park et al’s Park et al. (2023) Generative Agents. The main mode of validation in both papers was the ‘believability’ of the agents’ responses to a set of interview questions. Yu et al. (2024), however, offers no quantitative comparison between the two models, but merely includes the responses of agents, concluding that their version did not obviously impair the believability of the agents compared to the original.

#### Validation against human-generated data

A widely used validation technique involves comparing the output of generative agents to human-generated data, often through quantitative metrics. This approach is especially prevalent in simulations of social media environments, where models are evaluated against large-scale, real-world datasets.

Gao et al. (2023), Mou et al. (2024a), (2024b), Yang et al. (2024), Gu et al. (2025), and Ferraro et al. (2025) all validate their models by comparing synthetic data to content and behavior observed on platforms such as Twitter and Reddit. Common metrics include the speed and scale of information diffusion, user stances and attitudes toward specific events, and textual similarity between generated and real posts.

Outside the social media domain, Hua et al. (2023) use historical records to evaluate the plausibility of agent behavior in a geopolitical simulation—such as alliance formation and war declarations. Liu et al. (2024a), (2024b), (2024c) apply a similar approach using an Amazon product review dataset. They initialize agents using part of the purchase history and use the remaining data as ground truth to assess whether agents make similar purchase decisions.

Another common strategy is to replicate behavioral experiments previously conducted with human participants. For example, agents are tested in game-theoretic scenarios like the Trust Game (Xie et al. 2024) and the Ultimatum Game (Sreedhar and Chilton 2024), with results compared to known patterns of human strategic behavior. Chuang et al. (2024) validate agents’ performance in reproducing the “Wisdom of Partisan Crowds” effect, where exposure to group averages leads to belief convergence toward the ground truth.

Some studies rely on annotated human data to assess the accuracy of agent decisions. Wang et al. (2023) compare agent-generated emotion classifications to human annotations. Zhang et al. (2024a), (2024b), (2024c) evaluate agents’ choice of dialogue strategies against human-labeled ground truth. Jarrett et al. (2023) validate agent behavior as digital representatives by comparing outputs to a dataset of human opinions and critiques linked to demographic profiles.

#### Validation based on internal consistency

Some studies validate generative ABMs by examining their internal consistency, coherence, and stability—often through sensitivity analyses or controlled perturbations. The goal of such approaches is to ensure that agent behavior remains robust under varying conditions or parameter changes.

Hua et al. (2023), for instance, test the stability of their simulation by injecting counterfactual information and de-anonymizing countries to assess whether outcomes deviate in expected ways. De Marzo et al. (2023) vary the prompts given to their language model to determine whether the resulting network structure converges toward known forms, such as random graphs. He et al. (2024) conduct statistical tests on network metrics to validate the emergence of distinct communities, while Sreedhar and Chilton (2024) simulate agents with different personality profiles and assess whether resulting behavioral differences align with theoretical expectations.

While these forms of internal testing are relatively common, their interpretive power is limited. They may demonstrate that the model behaves in a logically coherent or parameter-stable manner, but this does not necessarily imply that the model provides a valid representation of real-world phenomena. As such, internal consistency tests are better viewed as a form of model verification rather than empirical validation.

## Discussion

Building on our review of how generative ABMs are employed and validated (RQ1 and RQ2), we now turn to RQ3: whether current validation strategies are sufficient to establish operational validity, and whether they address the long-standing challenges that have historically limited the uptake of ABMs in the social sciences.

Our analysis reveals some important signs of progress. Unlike classic ABMs, which often relied on informal plausibility arguments, roughly half of the reviewed studies incorporate some form of external, objective validation. This shift reflects a growing awareness within the field that credibility and scientific utility hinge on rigorous validation. However, these efforts remain uneven and have yet to arrive at reliable means of solving the fundamental challenge of establishing operational validity in ABMs.

A recurring weakness lies in the misalignment between the stated purpose of a model and the targets of validation. In several studies, validation focuses on surface-level outputs—such as stylistic realism of generated text—rather than the underlying mechanisms or interaction dynamics the model purports to capture. This form of weak coupling undermines the ability to evaluate whether the model faithfully represents the processes it aims to simulate.

While objective validation is becoming more common, subjective validation still remains dominant. 15 out of 35 studies rely solely on subjective assessments, and 22 use such assessments as their primary validation method. At best, these evaluations involve researchers or third-party raters assessing whether the simulation appears believable. At worst, validation consists of asking an LLM to evaluate the plausibility of its own output—a strategy that raises substantial concerns about circularity and bias. Recent work casts doubt on the reliability of LLMs for evaluation purposes, pointing to self-favoring tendencies and poor calibration (Wang et al. 2024a, b; Panickssery et al. 2024; Poláková et al. 2024). While some studies suggest that LLMs can match human raters in certain evaluation tasks (Chan et al. 2024; Zhou et al. 2024), this should arguably not be taken as a justification for LLM-based evaluation, but rather as a cautionary note about the limitations of untrained human judgment. Indeed, prior research shows that lay human raters struggle to reliably distinguish AI-generated from human-generated content (Clark et al. 2021). While subjective validation can offer a useful initial plausibility check, it does not, on its own, constitute evidence of operational validity.

When studies do carry out more rigorous objective comparison between human-generated and synthetic data, it often quickly becomes clear that the two are in fact substantially less similar than the face-validity believability tests may suggest. The style of writing, which tends to be the focus of such comparisons, between zero-shot LLMs and human conversations tends to be quite different: LLM responses are longer, more polite, articulate, and respectful (Zhang et al. 2024a, 2024b, 2024c; Mou et al. 2024a, 2024b). While humans may struggle to tell the difference, computational analyses tend to reveal substantial syntactical differences between human-generated and synthetic text (Pagan et al. 2025).

Yet, even studies that employ objective measures and empirical data rarely achieve operational validity. Validation must directly engage with the modeled mechanism in the context of the model’s intended application. For example, in simulations aimed at understanding algorithmic effects on social media interactions, validating linguistic style offers little support; more appropriate validation targets would include behavioral dynamics such as sharing behavior, exposure-response functions, or tie formation—depending on the mechanism under study. In short, validation must be aligned with purpose—not just demonstrate surface-level general notion of “realism.”

For models that aim to represent specific populations or demographic subgroups, some degree of profile alignment is also essential. While full individual-level calibration is typically infeasible, task-appropriate methods exist—for instance, linking agent attributes to survey or digital trace distributions, validating subgroup-level patterns, or testing whether the model reproduces known group disparities without reinforcing harmful stereotypes. Such calibration, however, remains underdeveloped and raises important ethical and methodological questions. In practice, nearly all current studies rely on zero-shot prompting without fine-tuning: “calibration” is thus reduced to prompt engineering, and generally aimed at improving face-validity. This creates substantial risks for LLMs misrepresenting social groups and reproducing problematic biases (Boelaert et al. 2025). While some papers acknowledge the presence of biases—such as the emergence of gender stereotypes in simulations (Xie et al. 2024)—few engage with the deeper question of how such biases affect the model’s capacity to reflect real-world behavior or inform policy-relevant insights. This suggests the need for studies to examine how rigorous calibration and validation of LLMs can be carried out to address such challenges.

Another challenge inherited—and exacerbated—from classic ABMs is computational cost. Traditional ABMs have long been criticized for their resource demands; generative ABMs, by comparison, are orders of magnitude more expensive. Even under conservative assumptions, token-based LLM pricing quickly leads to substantial costs. Consider a relatively simple setup with 100 agents, each engaging in 10 interactions per step over 100 steps, with 100 tokens of input and output per call. This amounts to 100,000 calls per run, or roughly 10 million input tokens and 10 million output tokens. At OpenAI’s lowest pricing tier as of June 2025 (GPT$$-$$4.1 nano: $0.10 per million input tokens and $0.40 per million output tokens), this translates into $1.00 for input and $4.00 for output—$5.00 in total for a single simulation run. While manageable in isolation, costs increase dramatically when scaling up. A modest two-parameter sweep with 10 values each and 10 repetitions per combination would require 1,000 runs, totaling $5,000. Using a more capable model such as GPT$$-$$4.1 ($2.00 per million input tokens and $8.00 per million output tokens) raises the price to $100 per run, or half a million dollars for the same parameter sweep. Since agent interactions scale quadratically with population size and computational demands increase exponentially with additional parameters, larger or more complex simulations rapidly become financially prohibitive.

Some papers have proposed techniques to mitigate these costs—for example, optimizing agent design (Kaiya et al. 2023; Yu et al. 2024), distributing computation (Yang et al. 2024), or hybridizing LLMs with rule-based agents (Mou et al. 2024a, 2024b). While these strategies help, they do not address the fundamental reality: generative ABMs are computationally intensive in ways that limit their scalability and accessibility, especially for researchers without institutional access to large-scale compute budgets.

A concerning expression of these costs is the widespread failure to conduct robustness checks or sensitivity analyses. Despite the well-known stochasticity of generative models, most reviewed studies rely on a single simulation run—akin to drawing general conclusions from a single-case study. This practice undermines the credibility of reported findings and limits the ability to assess the stability or generalizability of results. Without systematic exploration of parameter spaces or repeated trials, it is impossible to know whether observed outcomes reflect structural properties of the model or idiosyncratic artifacts of a single run.

In sum, the core challenges that have long haunted ABMs—their flexibility, which makes them powerful yet difficult to validate, reproduce, and compare—remain unresolved in generative ABMs. If anything, the introduction of LLMs exacerbates this tension. Although some studies recognize the absence of shared validation frameworks and benchmarks (De Marzo et al. 2023; Kaiya et al. 2023; Chen et al. 2024; Wu et al. 2024a, 2024b; Yu et al. 2024), and a few propose preliminary frameworks (Altera.al et al. 2024; Li et al. 2025; Chuang et al. 2024; Liu et al. 2024a, 2024b, 2024c), these efforts echo long-standing proposals in the ABM literature. Historically, such frameworks have seen limited uptake and have failed to constrain ABMs in ways that support comparability or cumulative knowledge-building. The greater complexity introduced by LLMs only heightens the difficulty of balancing standardization with the flexibility that makes ABMs attractive in the first place. While LLMs enable richer agent representations, they also impede the possibility of calibrating and validating agent behavior against real-world data.

## Conclusion

Generative ABMs expand the representational repertoire of social simulation by enabling agents that plan, reason, and converse in natural language. This paper has situated generative ABMs within the broader historical and methodological trajectory of agent-based modeling, and has examined whether and how the field is addressing the long-standing concerns that have historically limited the uptake of ABMs in the social sciences. Many of the reviewed studies are currently in preprint form, reflecting the rapid pace of development in this emerging field. We included these works due to their visibility and influence, as several are already widely citepd. However, it should be noted that revised versions may appear in peer-reviewed journals in the future, which may alter or refine their contributions.

Our review has revealed that generative ABMs are being applied to a wide variety of social phenomena—from network dynamics to online discourse—and that LLMs offer unprecedented opportunities for modeling complex, culturally embedded human behaviors. These models promise richer and more flexible representations of agency than conventional ABMs, with agents that go beyond deterministic rules or optimization routines.

Yet despite this promise, generative ABMs’ capacity to contribute to social science remain hampered by unresolved challenges of validity. As our review shows, integrating LLMs into ABMs does not, in itself, resolve these issues. On the contrary, it often exacerbates them: the opacity, stochasticity, and cultural biases of LLMs make empirical grounding and interpretability even more difficult to achieve. As a result, many studies rely on surface-level validation techniques, such as face-validity or plausibility assessments from single simulation runs.

The failure to convincingly show operational validity should not be understood as simply a matter of researcher oversight: calibration and validation represent fundamental unresolved challenges for generative ABMs. While such issues are acceptable in the early, experimental phase that characterizes of much of the current work, the key question is whether the field can evolve beyond such proof-of-concept models toward the kind of rigorous modeling that meaningfully contributes to social scientific theory. As we have argued, achieving this requires demonstrating *operational validity*—showing that the model plausibly captures underlying social mechanisms (Sargent 2010). In traditional ABMs, operational validity has typically been accomplished through one of two strategies.

The first strategy emphasizes empirical realism through careful calibration and validation (Edmonds and Moss 2004). At first glance, LLMs appear well-suited to this approach, as they can produce more human-like simulations of reasoning and communication. Yet the same factors that make LLMs appealing—complexity, adaptability, cultural embeddedness—also make them hard to calibrate, difficult to standardize, and potentially prone to reproducing social biases. As such, the empirical grounding of generative ABMs remains an unresolved challenge, and developing robust validation procedures for aligning LLM agents with real-world populations is an essential and ongoing task.

The second strategy is to construct stylized, simplified models that isolate core mechanisms and produce robust, generalizable insights. This approach, famously exemplified by the Schelling segregation model (Schelling 1971), treats ABMs as thought-experiments that illuminate fundamental social processes. These models are useful not because they mirror reality in detail, but *au contraire*, because their simplicity allows for transparent elucidation of emergent dynamics. Generative ABMs, however, pose a dilemma for this approach: their reliance on complex, black-box components makes it difficult to isolate mechanisms or trace causal pathways. As Axelrod notes, the strength of abstract modeling lies in the simplicity that enables making complex dynamics interpretable and explainable (Mandi et al. 2024; Axelrod 1997)—and the inherent lack of simplicity of generative ABMs means that they risk losing this explanatory clarity.

This places generative ABMs in an ambiguous methodological space between theoretical models and empirical data: they lack both the formal coherence and parsimony of abstract models and the empirical validity of data-driven approaches. As a result, it appears they can contribute neither theoretical insights nor empirical findings to social scientific knowledge.

For the field to move beyond proof-of-concept demonstrations, it must therefore clarify how these models can in fact contribute rigorous findings. Several paths forward are possible. One is to pursue more rigorous empirical validation to establish operational validity. The challenges involved in this has already been discussed. Another is to demonstrate context-specific generalizability—showing that generative ABMs can reliably produce valid insights in bounded empirical settings. A third is to develop techniques for enhancing interpretability, thus enabling generative ABMs to serve as effective tools for theoretical exploration of social phenomena.

Ultimately, however, it may be too limiting to judge generative ABMs solely by the standards of existing modeling paradigms. Rather than forcing these models into established methodological categories, it may be more productive to treat generative ABMs as a novel methodological genre. Their core strength may lie in an altogether new type of use, such as in their capacity to generate synthetic data or rapidly prototype ideas. In this light, generative simulations may be most productively used in early-stage theorizing—serving as creative, exploratory tools that complement rather than replace more conventional approaches in the social sciences. If these challenges can be met, generative ABMs may come to occupy a unique and valuable position in the methodological toolkit of the social sciences.

## Data Availability

No datasets were generated or analysed during the current study.
